# Supplementary material for: The emergence of hyper-altruistic behaviour in conflictual situations
Source: Sci Rep. 2015 Apr 28;4:9916. doi: 10.1038/srep09916 (PMC4412081; doi:10.1038/srep09916)
Supplement: Supplementary Information [file srep09916-s1.pdf]

Supplementary Information for "The emergence of hyper-altruistic behaviour in conflictual situations"

Valerio Capraro

Center for Mathematics and Computer Science (CWI), 1098 XG, Amsterdam, The Netherlands. Email: V.Capraro@cwi.nl

This Supplementary Information contains two sections. In the first section we provide all the details about the analysis of the free responses of the subjects who participated in our Study 3. In the second section we report the instructions used in our experiments.

Analysis of free responses

In order to support our conclusion that altruistic behavior in Study 3 was driven by hyper-altruistic subjects, that is subjects who evaluate other's payoff *strictly* more than their own, rather than by indifferent subjects, who evaluate the other's payoff the same as their own, we asked a research assistant to code each response from the altruistic participants in Study 3. The coder was not informed about the purpose of the study and the hypothesis and predictions being tested. For each statement, she was asked which of the following five categories best described it:

> The participant explicitly said that they took the action because that was the right thing to do (Rightness).

> The participant explicitly said that they took the action because the other action was wrong (Wrongness).

> The participant explicitly said that they took the action because they are generous (Generosity).

> The participant explicitly said that they took an action at random, because they were indifferent between the two actions (Indifference).

> The participant said something that is not classifiable in any of the previous categories (Not classifiable).

Below we report all 30 responses. Next to each response, in parenthesis, we report the category to which the response was assigned.

1. I'd feel bad if I took it (wrongness).
2. it's the holiday season..it is about giving, so I gave. I hope they appreciate it (generosity).
3. i would not feel right taking money from a person (wrongness).
4. I'm a giver, not a taker (generosity).
5. Did not want to be greedy. If there was a 3rd option of keeping my 10c and the other keeping his I would have selected that (indifference).
6. I like giving instead of receiving (generosity).
7. Rather have someone else gain a bonus (generosity).
8. Maybe the other person needs the money more than me and I won't take it from someone else's hand (rightness).
9. Just felt generous (generosity)
10. i just felt like being nice for once (rightness)
11. I wasn't going to take the other person's bonus (wrongness).
12. The other person most likely needs the money more than I do (rightness).
13. Although I considered taking the money, I decided that since it was such a small amount and that I would feel guilty for taking the money, I decided to give up my money (wrongness).
14. It's wrong to take something away from someone else for what amounts to an insignificant gain for myself (wrongness).
15. I would not feel good about myself by taking everything while another person had nothing (wrongness).
16. I thought it would make someone happy and they might need it more than I do (generosity).
17. I want to be good (not classifiable)
18. Better to give than recieve (rightness).

19. I am generous (generosity).

20. I didn't feel right taking all of the money (wrongness).

21. I'd rather give something than take something (generosity)

22. I would much rather give than take. It just doesn't feel right to take something away (wrongness)

23. I feel better not taking away the others money. The benefit is less than the cost of being mean (rightness).

24. I didn't think it was fair to take the other person's money (wrongness).

25. I felt that I wanted to give the amount of the bonus, because I felt it unfair to take all and assume that the other participant would gain more. I generally like to be fair, and hope that both participants receive an equal or substantial amount for the work done. I don't like to be overtly greedy (rightness).

26. Because I didn't want to take from the other participant, and since the only other option was to give my ten cents to him/her, I decided that worked better for my conscience (rightness).

27. It was the easiest choice and no conflict (not classifiable).

28. It was the most kind thing (rightness).

29. I had to make a choice (indifference).

30. I would have felt guilty leaving someone else with nothing but by giving up my 10 cents I feel as if I've done something small but good (wrongness).

Experimental instructions

The first screen, where the subjects were asked for their TurkID, was the same for all four studies. After this screen, participants entered the real game, where, in a single screen, we presented the problem and asked to make a decision. After making their decision, subjects entered a standard demographic questionnaire where we asked for their gender, age, education level, and reason for their choice (only in Study 1 and Study 3). Below we report the instructions used in the decision screen for each of the four studies. For each study, we report only the instructions used in the no-exit condition. Those for the other conditions were very similar, a part from the obvious changes.

Study 1

You have been paired with another anonymous participant. You both own $0.30 for participating in this HIT. Please choose one of the following alternatives:

- Donate your $0.30 to the other participant. In this case you end the game with nothing and the other participant ends the game with $0.60.

- Steal the participation fee from the other participant. In this case you end the game with $0.60 and the other participant with nothing.

Study 2

You have been paired with another anonymous participant. You are both given additional $0.30 as a bonus. Please choose one of the following alternatives:

- Give your $0.30 to the other participant. In this case you end the game with nothing and the other participant ends the game with $0.60.

- Take the other participant's bonus. In this case you end the game with $0.60 and the other participant with nothing.

Study 3

You have been paired with another anonymous participant. You are both given additional $0.10 as a bonus. You can either give your $0.10 to the other participant or take his or her $0.10. In this latter case, the money will be doubled and earned by you. What is your choice?

- Give your $0.10 to the other participant. In this case you end the game with nothing and the other participant with $0.20.

- Take $0.10 from the other participant. In this case you end the game with $0.30 and the other participant with nothing.

Study 4

You have been grouped together with other two participants, Person A and Person B. You are all given additional $0.30 as a bonus. Please choose one of the following alternatives:

- Take the $\$0.30$ from Person A and share them with person B. In this case, Person A will finish this task with $\$0$ and you and Person B will finish this task with $\$0.45$.

- Take the $\$0.30$ from Person B and share them with person A. In this case, Person B will finish this task with $\$0$ and you and Person A will finish this task with $\$0.45$.

- Give your $\$0.30$ to Person A and Person B. In this case, you will finish this task with $\$0$ and Person A and Person B will finish this task with $\$0.45$.
